# Tie and tag: A study of tie strength and tags for photo sharing

**DOI:** 10.1371/journal.pone.0202540

**Published:** 2018-08-29

**Authors:** Ricard L. Fogues, Jose M. Such, Agustin Espinosa, Ana Garcia-Fornes

**Affiliations:** 1 Departamento de Sistemas Informaticos y Computacion, Universitat Politecnica de Valencia, Spain; 2 Department of Informatics, King’s College, London, United Kingdom; Dalian University of Technology, CHINA

## Abstract

Tie strength and tags have been separately suggested as possible attributes for photo access controls in Social Network Services. However, an evaluation is missing about the benefits/drawbacks of adding one or both of these attributes to the ones already used in access controls for Social Network Services (groups and individuals). In this paper, we describe an experiment with 48 participants using access controls that include tie strength and tags (separately and simultaneously) together with attributes for groups and individuals. We analyze the results using several quantitative and qualitative metrics. We find that users consider these two new attributes useful in defining their sharing policies, and they prefer to employ access controls that consider tags and tie strength jointly. Specifically, users believe that tie strength improves policy understandability and that tags help them define sharing policies faster. However, we also observe that when users employ these two attributes they tend to make more mistakes in terms of the resulting sharing policy. We hypothesize that this could be caused by the lack of experience using tie strength and tags in access controls.

## 1 Introduction

Millions of photos are shared everyday on Social Network Services (SNSs) such as Facebook, Instagram, and Google+. SNSs enable users to set sharing policies to control how photos are shared with other users. These sharing policies are defined as rules that specify who can access a photo. The *who* can be set by employing two elements: groups and individual identifiers. Groups can be defined by each user (e.g., Google+ circles or Facebook friend lists) or they can be predefined by the SNS (e.g., friends of friends). These two elements (groups and individual identifiers) allow definitions such as “share this photo with everyone but Bob”.

As defined by Nissenbaum [1] in the contextual integrity framework, personal information moves from one context to another based on norms of information flow. When one of these norms is violated, a privacy breach occurs. When SNS users define their sharing policies, they are basically defining their norms of information flow. However, as a number of studies have shown, it is difficult for SNS users to set their sharing policies appropriately using access controls with only groups and individuals as attributes [2, 3]. Consequently, current access controls may not be appropriate to guarantee that the personal information of SNS users flows only through contexts that they consider appropriate.

We identify two key elements of the contextual integrity framework that current access controls for SNS are lacking: tie strength and content type. Nissenbaum points out that distinctive relationships (e.g., individual to spouse, boss, friend, colleague, etc), are partially defined by distinctive patterns of information sharing. This was already studied in off-line social networks by Granovetter in 1973 [4]. Research works on social media have looked into how people share with contacts of varying tie strength (e.g., [5]). The results presented in these works show that the tie strength of a relationship is an important factor that users consider when deciding whether or not disclosing information is appropriated.

As explained by Nissenbaum, the definition of norms of information flow is based on the type of the information. For example, people usually do not share health and medical information with strangers. Since we focus our research on photo sharing, we propose the use of tags to define the content of the photo. Currently, SNSs do not allow users to define sharing policies based on the content of the information. However, some photo sharing sites, such as Flickr, enable users to assign tags to their photos so they can be found and classified easily.

Our key objective in this paper is to evaluate the actual impact that tags and tie strength would have on access controls for SNSs. We have designed a study where 48 Facebook users employ different access controls to express their privacy preferences on a selection of 30 photos of their own. With the data collected in the study, we evaluate the impact of tie strength and tags on access controls for photo sharing. This evaluation is based on both the quantitative measures of their performance and a qualitative analysis of the participants’ self-reported perceptions.

### Contributions

Our contribution is threefold:

1. We evaluate four attributes for access controls: groups, individuals, tie strength, and tags.
2. To evaluate the attributes and the access controls that use them, we propose novel quantitative metrics.
3. We present a qualitative evaluation of tie strength and tags as attributes for access controls. Additionally, by using thematic analysis on the qualitative data, we identify three characteristics that users value from access controls.

## 2 Related work

A number of papers propose formal access control models for SNSs [6–8]. Some of these models consider the inclusion of tie strength as an attribute, but none of them consider tags. The authors of these works only focused on the expressiveness of the proposed models. Our work investigates the viability of including tie strength and tags and how they affect access controls.

Several papers point out the importance of the content to information flow [9–11]. Tagging data is a method for identifying the content of that data. Access controls for photos that take tags into account have been prototyped by Yeung et al. [12]. Besides, Hart et al. [13] proposed a mechanism to manage privacy for blogs based on tags. Their mechanism enables users to define groups manually or to group potential viewers by attributes that they all share (e.g., workplace or same school). The main focus of their study is to compare basic sharing policy mechanisms for blogs with a tag-based approach. The authors did not use real data from the participants; instead, they asked participants to manage sharing policies of artificial users in hypothetical scenarios. Thus, they do not examine users’ actual preferences. Their results show that an approach that uses tags is more appealing to users than one that does not. Klemperer et al. [14] evaluated the usability of an access control based exclusively on tags. The authors aimed at evaluating whether tags can be used to organize pictures and define their privacy at the same time. However, we study the viability of including tags as a new attribute for access controls along with other attributes that already exist (groups and individual identifiers) and a new one, tie strength.

Several research works focus on the imagined audience that people have in mind for the information they share on SNSs [15–17]. As shown by these papers, people aim at reaching certain audiences for each post they share. However, as shown by Litt and Hargittai [18] the imagined audience for each post published by a user on SNSs fluctuates among several target audiences even though the user does not change the sharing policy from post to post. This shows that selective sharing, although rare, is necessary to harmonize the imagined and potential audience of the information shared on SNSs. One of the reasons why people do not tailor sharing policies to match their imagined audiences is the lack of tools to easily manage sharing policies. This forces users to employ a variety of *coping strategies* [19]. For example, self-censorship or *unfriending* contacts. However, these strategies have limited effectiveness in practice.

Besides the complexity of access control [20], setting sharing policies can be a tiresome task. Sometimes, this complexity deters users from employing specific sharing policies for each item individually. To help users cope with this task, a number of privacy recommender tools have been developed [21–26]. These tools alleviate the burden of privacy configuration in SNSs. The tools proposed in these works aim at facilitating privacy configuration in current access controls. Therefore, they take into account the same attributes of these access controls. We believe that an SNS that offers a combination of access controls that employ attributes that help users understand the implication of privacy settings and privacy recommenders will enhance users’ experience.

## 3 Method

We designed a laboratory study during which participants used different access controls to specify sharing policies for photos of their own. By observing and analyzing how the participants defined their sharing policies, we can evaluate the performance of tie strength and tags as attributes for access controls. Moreover, the participants provided their personal impressions about these two attributes. This qualitative data helps us to test whether users perceive the inclusion of these new attributes as beneficial for managing their privacy on social media.

Our investigation is based on real data that was collected from Facebook users. We specifically chose Facebook users for two reasons: (i) Facebook users are accustomed to sharing information on the Internet and using sharing policies to protect it; and (ii) Facebook is one of the most successful SNSs, so there are many potential participants.

To recruit participants, we published the study on a number of social media sites. We relied on the viral properties of social media to attract diverse participants outside of the academic environment. Therefore, as people signed up for the study, we asked them to share their participation on their social media accounts. To reward the participants for their time and effort, we gave them a 10-Euro gift voucher.

Since the information required from the participants was varied, we divided the study into six steps. In each step, the participants provided a different type of information. The steps were: (i) collection of social data, (ii) choosing photos, (iii) tagging photos, (iv) definition of sharing policies, (v) correction of sharing policies, and (vi) post-survey questionnaire. These steps are explained in further detail in the following sections. The average time to complete the study was around one hour and a half. Since completing the study in one session could be tiresome, it was possible to stop and resume it at any time.

During the study, the participants provided personal photos, and we collected sensitive data from their Facebook accounts. We informed them beforehand that their collected data would only be used for academic purposes. Moreover, we committed to anonymizing their data as soon as they finished the study. Likewise, the participants were informed that the photos used during the study would not be seen by any member of our group at any time and that we would delete them as soon as they finished the study. The participants provided a written consent so we could collect and process their personal information. We also obtained approval for our study from the ethics committee at Universitat Politecnica de Valencia; approval was made in writing.

### 3.1 Demographics

The sample consisted of 23 women and 25 men (48 total). With regard to age, 52% were between 18 and 24 years old, 23% between 25 and 29, 21% were between 30 and 39, and 4% were between 40 and 49. With regard to education level, the majority of them had a college degree (23), 20 of participants had a high school degree, three of them had a PhD, and two participants had a primary school degree. Finally, 72% of the participants were students and the other 28% were working.

### 3.2 Step 1: Collection of social data

During the evaluation, we use data that was collected from the participants’ Facebook accounts. Specifically, their relationship information (i.e., how they group their contacts on Facebook and tie strength values). To collect this information, we used BFF [27]. This is a Facebook application that helps users organize their relationships. BFF automatizes the process of friend grouping and tie strength definition. To calculate tie strength values, BFF utilizes a linear model that employs a variety of predictors such as number of common friends, or *likes* given. To create groups, BFF uses Infomap community finding algorithm [28]. BFF represents tie strength on a Likert scale (1-5: 1 = minimum, 5 = maximum). Therefore, we used the same level of granularity to represent tie strength levels.

BFF was only used as a way to collect data from Facebook and to speed up the process of defining the characteristics of the social relationships of the participants. Based on previous research, BFF achieves an accuracy of over 80% for tie strength prediction. The results of BFF could be modified as the participants considered necessary. It is worth noting that, like in Facebook, contact groups were not exclusive; therefore, one single contact could be included in several groups if the participant found it appropriate.

**Fig 1:**
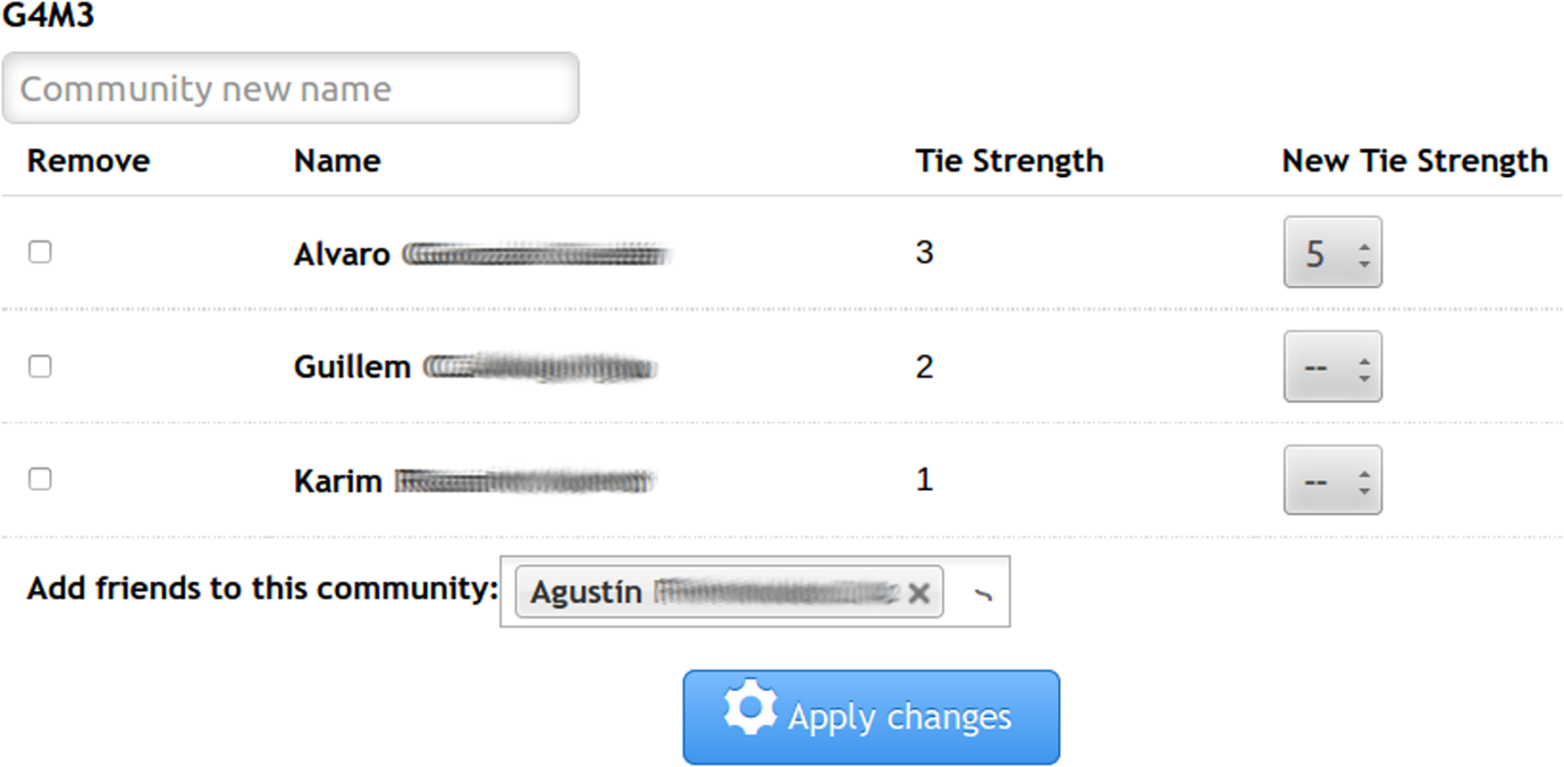
Tie strength and group correction interface.

Fig 1 shows the user interface that presents the results obtained by BFF and offers the possibility to the user to adjust them. In the example shown in Fig 1, the user is adding a new member to a group and changing the tie strength of a contact (*Alvaro*) from 3 to 5.

As shown in [14], when people use tags to specifically manage their privacy, they behave differently to when they use the tags as a means to organize their photos. Likewise, users can be expected to employ other attributes, such as tie strength, differently if they are told beforehand that they will be using those attributes for privacy management. Since we wanted to observe how users manage their sharing policies employing the different attributes, the participants were instructed to group their contacts, tag their photos, and assign tie strength values taking into consideration that they would be using that information to define their sharing policies.

### 3.3 Step 2: Choosing photos

**Fig 2:**
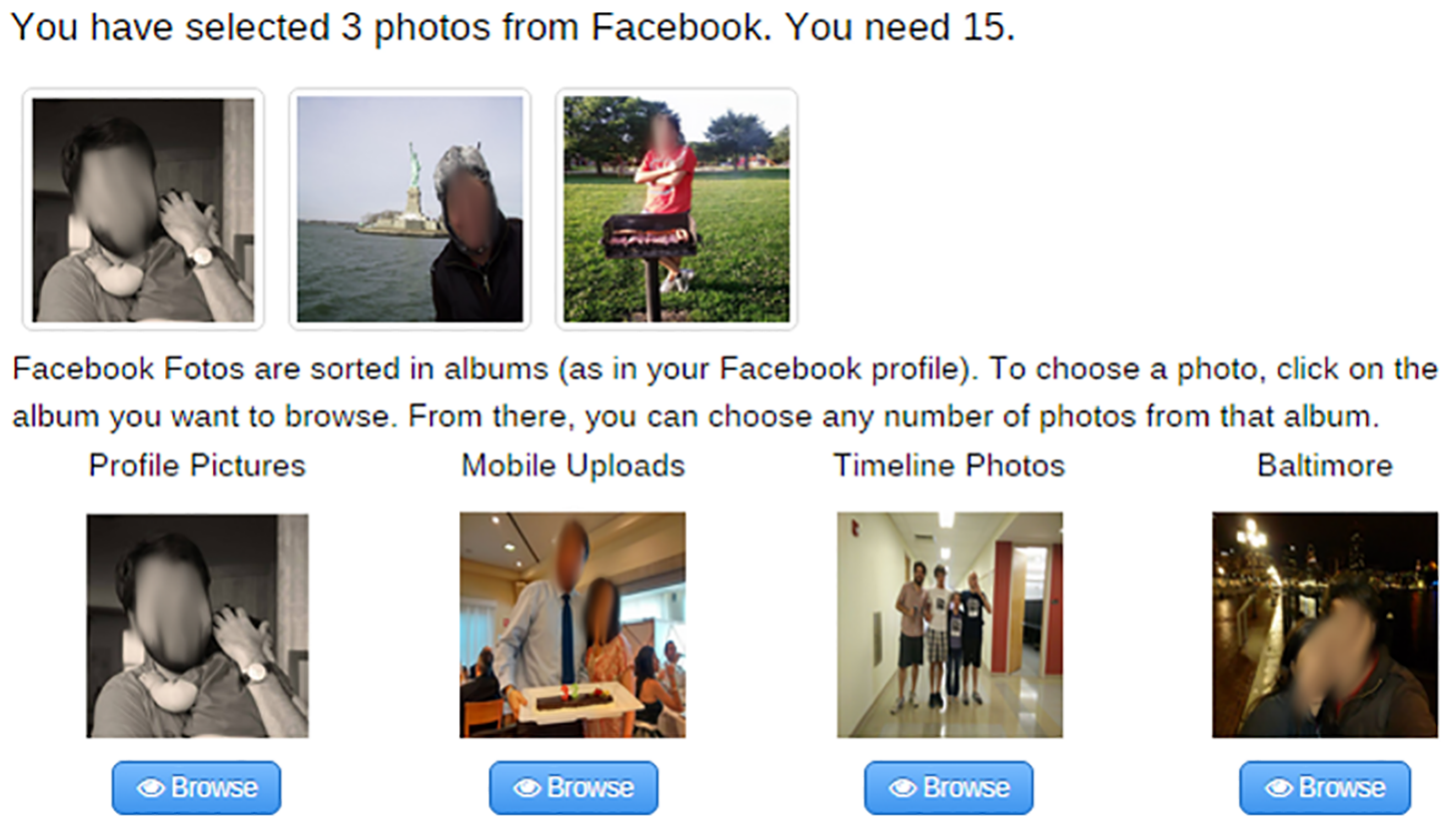
Photo selection interface.

Since we wanted to collect information on how users manage the privacy of their photos in a variety of situations, we asked the participants to choose 15 photos from their Facebook accounts and 15 photos that they had not shared on any SNS. The idea behind this was to try to collect photos that the participants did not share on any SNS due to privacy concerns. In this way, we can evaluate new access controls and attributes on photos that users believe current access controls do not protect well. We instructed the participants to choose 15 photos from outside of Facebook that were suitable to be shared with at least one contact on Facebook, even though they were not shared on any SNS. In this way, we prevented the participants from choosing photos that were so sensitive that their only appropriate sharing setting was to keep them private. Additionally, we asked participants to choose photos that depicted specific items. We brainstormed a list of 17 items, which included elements such as selfies, friends, special days, kids, a moment with your significant other, and food. The participants were not required to cover every item, but we asked them to cover as many as possible. The goal of this requirement was to force the participants to choose photos with the widest possible variety. Fig 2 shows the interface used by the participants to choose their 30 photos. In the example, the participant has so far chosen three photos from Facebook and none outside of Facebook. It is worth noting that all of the photos shown in the examples are mockups and not real photos used in the study.

### 3.4 Step 3: Tagging photos

**Fig 3:**
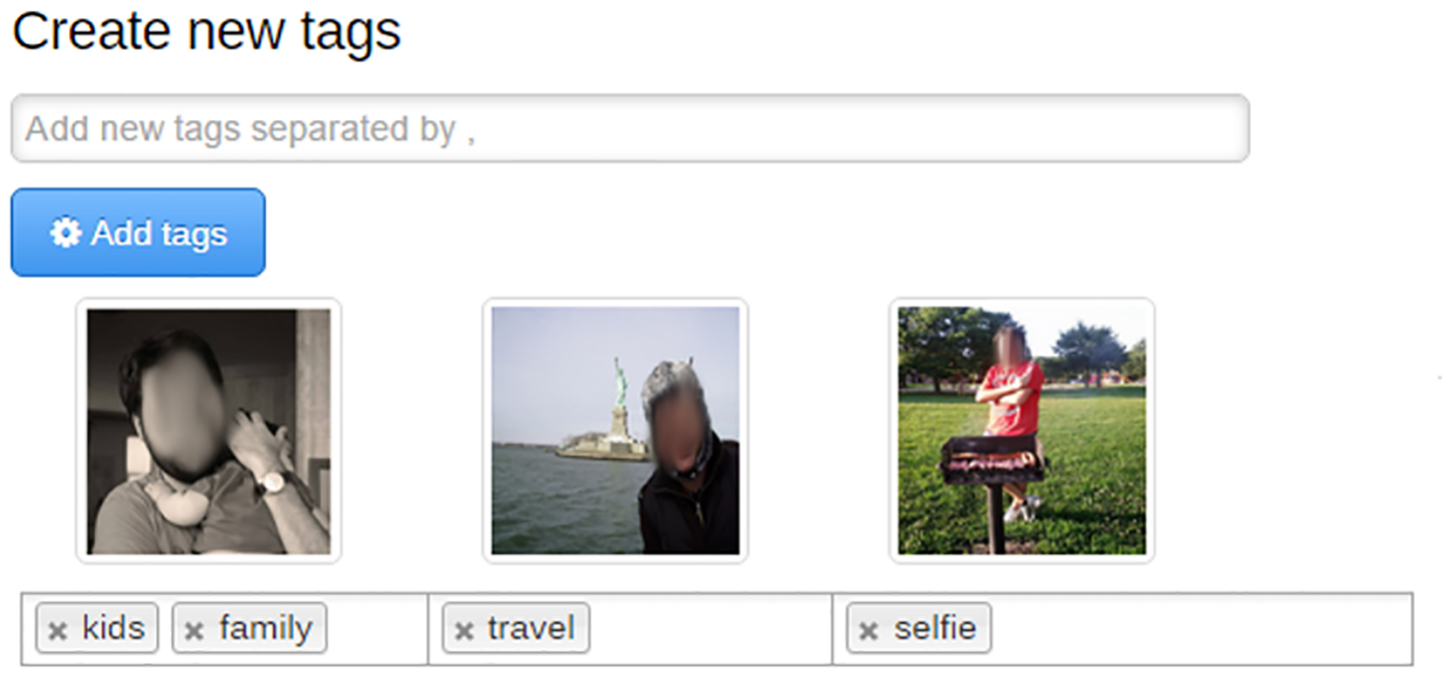
Assigning tags to photos.

After the participants chose their photos, they had to assign tags to them. The tags were free and each participant defined as many as he or she felt necessary. Each photo had to be classified with at least one tag and a photo could be classified by using many tags. Fig 3 shows the interface that the participants used to assign tags to their photos. In Fig 3, the shown photos have been tagged as *family* and *kids*, *travel*, and *selfie*, respectively.

### 3.5 Step 4: Definition of privacy preferences

In this step, the participants had to specify their privacy preferences using policies defined with two different access controls. These access controls had different combinations of the following attributes:

- **Tags (*Tag*)**: Labels or categories that users employ to organize their photos by their content (e.g., family, and travel).
- **Groups (*Group*)**: Groups of contacts created by the users.
- **Individual Contacts (*Ind*)**: Individual identifiers of each contact of the user.
- **Tie Strength (*Tie*)**: The level of closeness that users have with each one of their contacts based on a Likert scale from 1 to 5 (1 = weak tie, 5 = strong tie).

Employing these attributes, we defined three different access controls: TagGroupIndTie, GroupIndTie, and TagGroupInd. We name the access controls based on the abbreviated names of the attributes that each one uses. For example, GroupIndTie employs groups, individual contacts, and tie strength. We employed these specific three access controls because they enable us to compare (together and separately) tie strength and tags with groups and individuals. The most direct approach for collecting data to compare all of the access controls would have been to ask the participants to express their privacy preferences using all of the access controls. This approach was not feasible as it would have made the study too demanding. The participants would have been required to assign a sharing policy to each photo three times (one for each access control). This may have fatigued them, which, in turn, may have led to sloppy responses. Instead, we asked the participants to set their privacy preferences using only two of the access controls. In this way, they could compare the benefits and disadvantages of one combination of attributes over the other.

**Fig 4:**
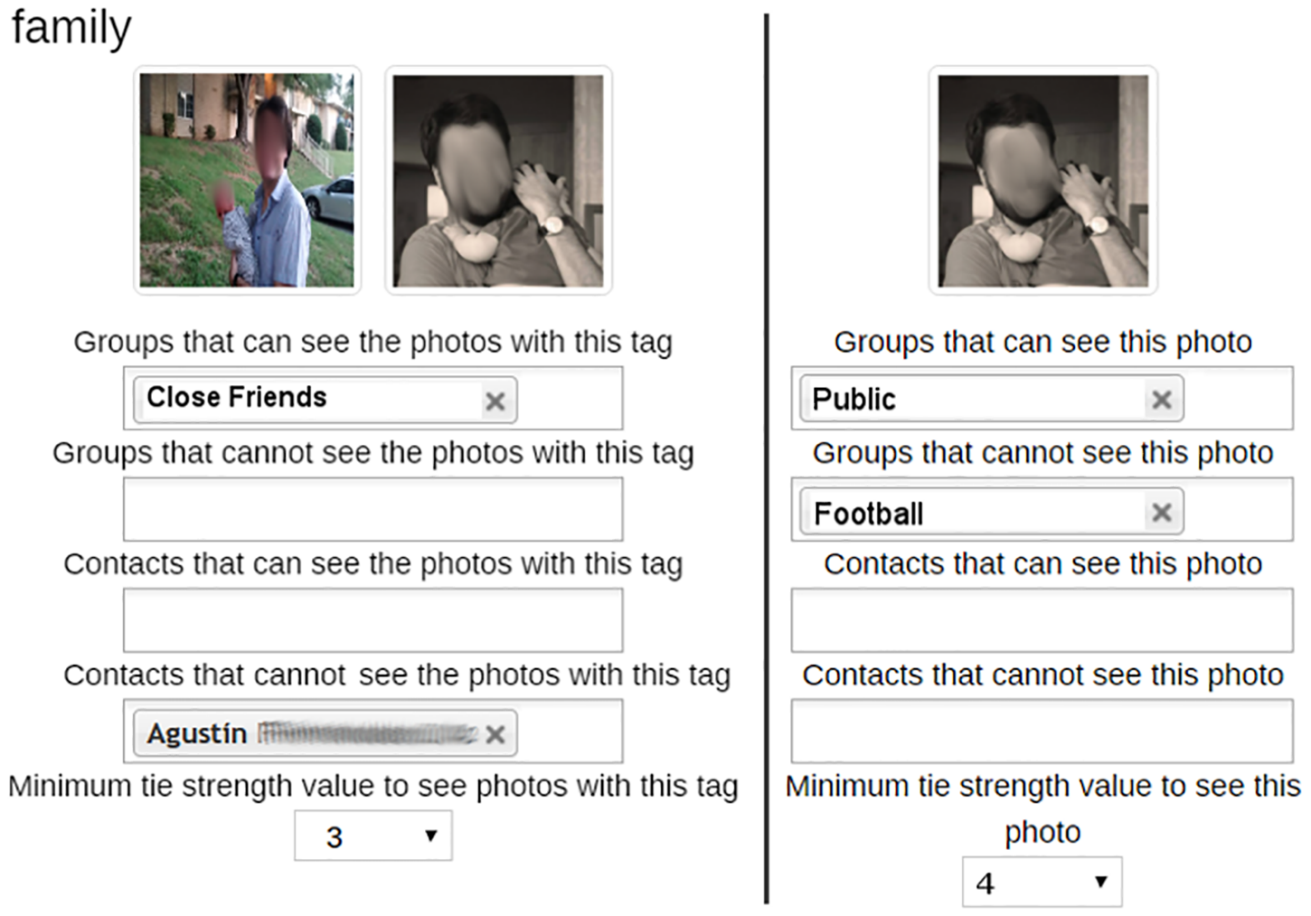
Sharing policy definition (tag view on the left and photo view on the right).

Fig 4 shows the interface that the participants used to define their sharing policies. While using TagGrouIndTie or TagGroupInd, the participants used an interface to define sharing policies that offered two views: photo and tag. The photo view enabled the participants to define their sharing policies on a per photo basis. On the other hand, the tag view enabled them to define sharing policies on a per tag basis. These two views were accessible through tabs. Fig 4 shows the tag view on the left and the photo view on the right.

Regardless of the access control, sharing policies were defined in terms of two predicates: allow and block. Contacts that are referenced by any attribute in the allow predicate are granted access to the photo (or photos with a specific tag), and vice versa for the block predicate. Following the same approach as mainstream SNSs, block takes precedence over allow. The tie strength attribute worked slightly differently. The participants using either TagGroupIndTie or GroupIndTie could specify a tie strength threshold in their sharing policies. All contacts with a tie strength value equal to or higher than the threshold were granted access to the photo or tag; the others were blocked. Finally, by default, no contact had access to any given photo; however, the default group *Public* includes all contacts. Using the photos shown in Fig 4 as an example, on the one hand, the photos tagged with *family* (on the left) can be seen by the members of the group *Close Friends*, excluding Agustin and all those who have a tie strength value lower than 3. On the other hand, for the photo on the right, it can be seen by everyone (*Public* group) except members of the *Football* group and contacts with a tie strength value lower than 4. All this was explained to the participants before they started the study, and they were reminded of this during this step.

We collected the same number of samples for every access control; specifically, each access control was employed 32 times (48 participants × 2 access controls ÷ 3 access controls). Moreover, the order of how the access controls were presented to the participants was random. This measure was taken to counteract any ordering bias (e.g., participants getting used to the interface of the survey application), thus, finding themselves more comfortable with the second access control. Finally, we also randomly assigned the order in which tabs for tags and photo views were shown. In this way, we also removed any bias generated from showing any of those views first.

### 3.6 Step 5: Correcting sharing policies

After the participants defined their sharing policies using both access controls, the next step was to correct errors in the sharing policies. The participants were using new access controls with attributes that they had not used before. Therefore, some mistakes could be expected. Additionally, the participants defined their sharing policies using two different access controls, so divergences between sharing policies for the same given photo were likely to occur.

**Fig 5:**
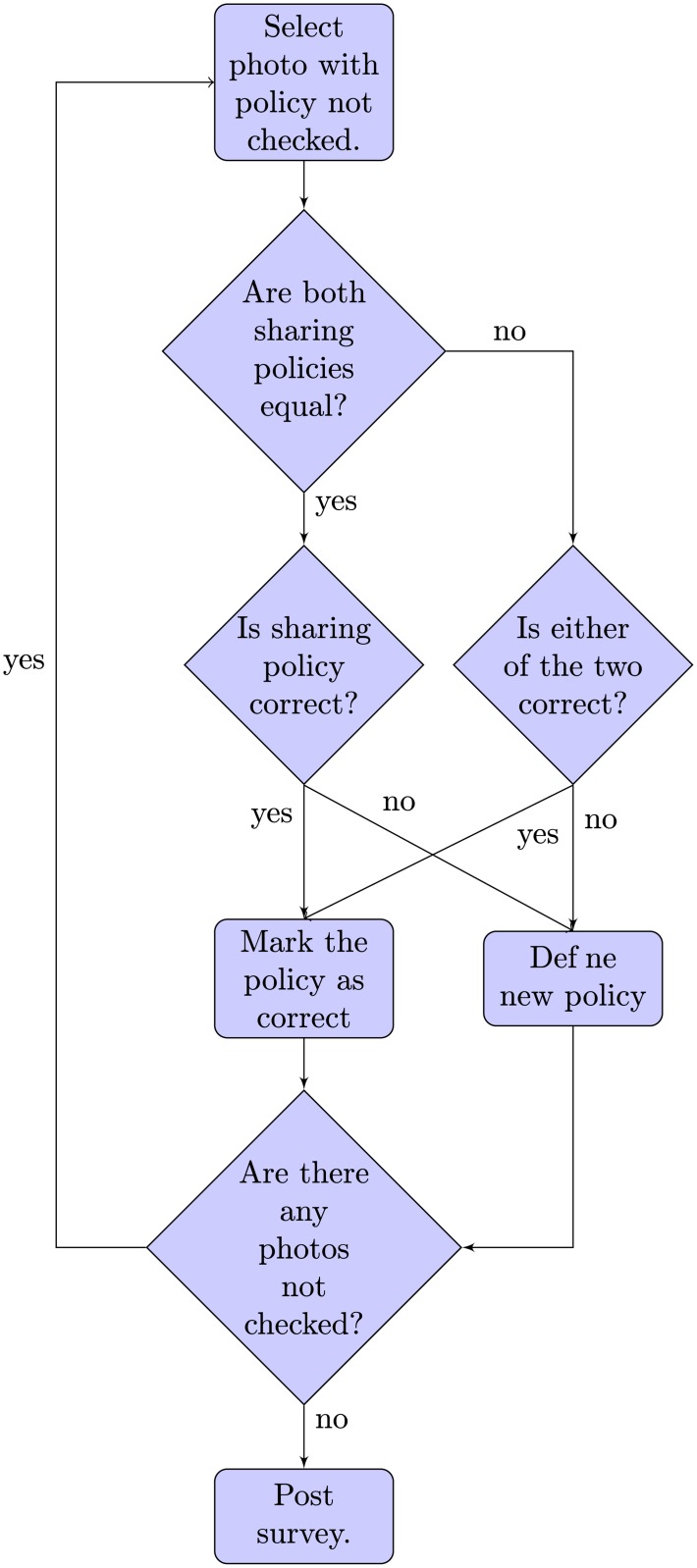
Procedure for correcting sharing policies.

Since the participants used two access controls, each photo had two associated sharing policies. In this step, each photo was shown in conjunction with the list of contacts that were allowed and not allowed to see it according to each policy. In this way, the participants could thoroughly review the sharing policies they had defined in the previous step. When the two sharing policies for the same photo (one for each access control) were the same, the participants could choose to mark them as correct or mark them as incorrect and modify them. On the other hand, if the policies were different, participants could either choose one as correct or neither. If they did not chose one, they had to modify one of the two. Fig 5 shows a flow chart that represents the procedure that the participants followed to correct their sharing policies.

**Fig 6:**
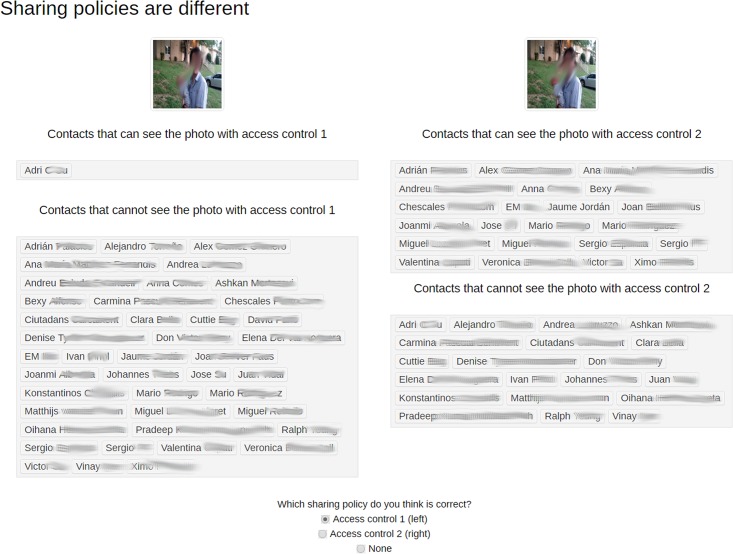
Correcting sharing policies.

Fig 6 presents the webpage that shows the sharing policies defined for a single photo with the two access controls. In the example shown, the two sharing policies are different. Therefore, the participant is asked to choose if one is correct or neither is. In the case neither is correct, the participant has to correct one of the two policies and mark it as correct.

### 3.7 Step 6: Post-survey questionnaire

In the last step of the study, the participants responded to a survey about the access control they liked the most. The participants were required to articulate their decision as much as possible. They also reported some information about privacy practices and how they use Facebook (see S1 Appendix for the original version of the questionnaire in Spanish, and S2 Appendix for the translated English version). Specifically, they reported the following data: age of Facebook account, frequency of use of Facebook, number of photos on Facebook, level of privacy concern while using social media, whether or not they changed the default sharing policies of Facebook, whether or not they created one friend list on Facebook, level of knowledge about Facebook’s use of personal data policy, and how often they specify a sharing policy while sharing a photo on Facebook.

## 4 Evaluation

In this section, we describe our hypotheses and evaluate them. The evaluation is based on the sharing policies defined by the participants. We consider each sharing policy defined by the participants as one sample. Since each participant had to define the sharing policy of 30 photos using two access controls, the dataset contains 2880 samples (48 participants × 30 photos × 2 access controls).

### 4.1 Hypotheses

The hypotheses tested in this paper are:

- *H-Useful-Tie-Strength*: Users find tie strength useful to define sharing policies.
- *H-Useful-Tags*: Users find tags useful to define sharing policies.
- *H-Performance-Tie-Strength*: Users can accurately express their privacy preferences when they employ sharing policies that use tie strength.
- *H-Performance-Tags*: Users can accurately express their privacy preferences when they employ sharing policies that use tags.
- *H-Preference-Tie-Strength*: Users prefer to employ access controls that include tie strength.
- *H-Preference-Tags*: Users prefer to employ access controls that include tags.

### 4.2 H-Useful-*

To evaluate these hypotheses, we measure the usage of each attribute in the policies defined by the participants. We count as one use of an attribute to specify one or more values for that attribute. For example, the policy *Allow*{Group(friends), Alice}, *Block*{Group(colleagues)} uses the attributes group and individual. The usage of an attribute is the average number of times that the given attribute was employed by a participant to define a sharing policy using an access control that considers that attribute. For instance, if a participant using GroupIndTie employed group to define the sharing policies of 15 of his 30 photos, the usage of group for that participant would be 50%. Our intuition is that the more a user employs an attribute, the more he/she feels it is useful to define sharing policies.

**Fig 7:**
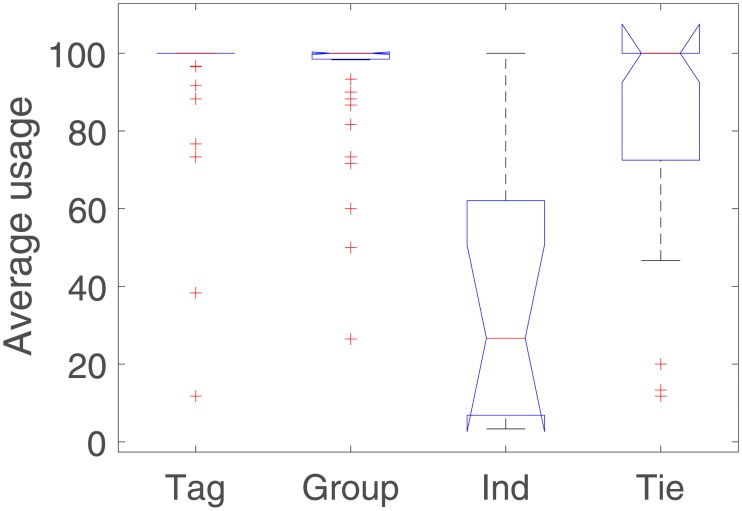
Average attribute usage per participant.

Fig 7 is a boxplot that shows the average usage of each attribute per participant. The line inside the boxplot indicates the median. The tops and bottoms of each “box” are the 25th and 75th percentiles of the samples, respectively. Whiskers are drawn from the ends of the interquartile ranges to the furthest observations that are not considered to be outliers. An outlier (represented as a cross) is a value that is more than 1.5 times the interquartile range away from the top or bottom of the box. Finally, the notches of the boxes display the variability of the median between samples. Boxes whose notches do not overlap have different medians at the 5% significance level.

On the one hand, tag, group, and tie are intensively used and their differences in use are not statistically significant; however, tie strength presents a higher variance. On the other hand, individual is much less used than the other three attributes. These results indicate that users find tag, group, and tie strength to be more useful than individual identifier.

### 4.3 *H-Performance*-*

To evaluate H-Performance-* hypotheses, we build *regression* models with multiple predictors and one response variable. The predictors are dummy variables that represent whether or not an attribute was used in a given sharing policy. For example, the policy *Allow*{Group(friends, family), Alice}, *Block*{Group(colleagues)} uses the attribute group and the attribute individual identifier. Therefore, in this example, the variables *group used* and *individual used* equal 1, while *tag used* and *tie strength used* equal 0. These dummy variables are used as predictors in the model. When analyzing the output of the model, we focus on *coefficients* and their statistical significance. These coefficients help us understand each feature’s relative influence on the response variable.

Since each participant defined several sharing policies, each participant’s personal experience with access controls may affect the estimated coefficients of the models. Thus, we employ mixed modeling [29] to create all of the regression models. Mixed models offer the possibility of grouping samples of the data hierarchically. Specifically, we group sharing policies by participant by using the participant ID as a *random effect*. In this way, the participant ID captures the variability introduced by personal experiences with access controls in the responses. Introducing the ID enables us to obtain estimated coefficients for the other predictors (*fixed effects*) in a way that is less affected by the variability in the participants’ responses. Finally, to show the significance of the coefficients, we use * and ** to indicate p<0.05 and p<0.01, respectively.

The first model is a *logistic regression model* where the response variable is categorical and indicates whether or not the policy is correct. Since the participants had to review and mark each policy as correct or incorrect, the computation of this response variable is straightforward.

In logistic regression models, the coefficients are expressed in log-odds units. The coefficients show the effect of a predictor on the log odds of the sharing policy being in a given category (*Y* = 1) versus being in the reference category (*Y* = 0); that is, the odds of *Y* being a given category increase by a factor of $e^{b_{i}}$ per unit change in *X*_*i*_. The equation used by the logistic models is

$$\begin{array}{c} P\left(Y=1\right)=\frac{1}{1+e^{-b_{0}-\sum b_{i}X_{i}}} \end{array}$$ (1)

where *b*_*i*_ is the coefficient of predictor *X*_*i*_.

Table 1 shows the output of the multinomial logistic regression. Our intuition is that an attribute with a negative coefficient indicates a disconnection between how the user believes the attribute works in terms of blocking and granting access to contacts and how it actually does. Specifically, using tags increases the risk of defining an incorrect policy, while using groups and individual identifiers reduces the risk. The influence of tie strength on the probability of a policy being correct/incorrect is not significant.

**Table 1:** Coefficients for the policy correctness regression model.

	Correct = True
**Tag Used = True**	−0.528**
**Group Used = True**	−0.745**
**Ind Used = True**	−0.402
**Tie Used = True**	−0.079

The logistic regression model assumes that each predictor is independent. However, in the study, the participants defined policies combining different attributes. Therefore, this assumption may not be true. To study how the combination of attributes affects the probability of a policy being correct, we create a new logistic regression model with the access control employed to define the policy as the predictor variable. Table 2 shows the coefficients of each access control (GroupIndTie is the reference category; hence, it is not shown in Table 2). The coefficients indicate that a policy defined with TagGroupIndTie or TagGroupInd has a higher risk of being incorrect than one defined with GroupIndTie.

**Table 2:** Coefficients for the policy correctness regression model employing access controls as predictors.

	Correct = True
**Access Control = TagGroupIndTie**	−0.867**
**Access Control = TagGroupInd**	−0.379*

**Fig 8:**
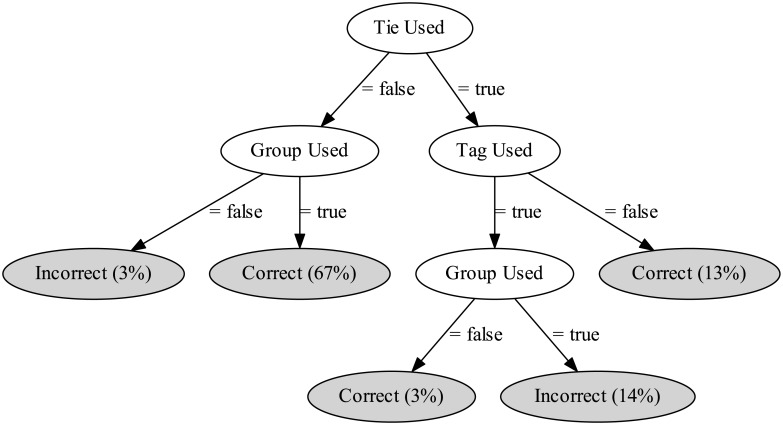
A decision tree with the correctness of policies as decision targets (leaf nodes; shaded).

To further explore the relationship between policy correctness and attribute combinations, we build a C4.5 decision tree [30] using Weka implementation [31], which achieves an accuracy of 74.4%. Fig 8 shows the resulting decision tree, where each leaf represents a class (i.e., correct or incorrect) and shows its name and the percentage of instances reaching that leaf. Our objective in training the decision tree is to interpret the paths in the tree. Our intuition is that the path from the root to a leaf in the tree describes the combination of attributes used that leads to a sharing policy being correct or incorrect.

We observe that the way tie strength is combined with other attributes is crucial for the correctness of a policy (tie strength appears in the root node of the tree). On the one hand, if tie strength is combined with groups and tags simultaneously, incorrect policies can be expected. On the other hand, if tie strength is not employed, or employed without tags, the chances of the policy being correct increase.

The models created with the correctness of the policy as the response variable provide a measure of the influence that attributes have on defining an incorrect policy. However, they do not measure the influence of the attributes on the seriousness of the privacy breach produced by the incorrect policy. For example, the privacy breach produced by a policy that incorrectly allows access to a single contact is likely less serious than the breach produced by a policy that incorrectly grants access to dozens of contacts. To this aim, we calculate the mutual information between each policy marked as incorrect by the participants and its corresponding corrected one. The mutual information is in the range [0, 1]; it equals 0 when both policies (the incorrect and the correct) are completely disjoint, and it is close to 1 when the differences are minimal. Since the mutual information is a continuous variable, we build a *multiple linear regression model* with the mutual information as the response variable. Linear regression models use the general linear equation

$$\begin{array}{c} Y=b_{0}+\sum b_{i}X_{i} \end{array}$$ (2)

where *Y* is a continuous response variable and *b*_*i*_ is the coefficient of predictor *X*_*i*_. As in the logistic regression models, we employ mixed modeling to reduce the influence of personal experience with access controls on the coefficients. Table 3 shows the coefficients yielded by the linear regression model. Analyzing the model output, high coefficients indicate that the attribute contributes to reducing the privacy breach caused by an incorrect policy. Specifically, individual identifiers is the attribute that reduces privacy breaches the most, followed by tie strength. Using groups and tags is not statistically significant. Therefore, when users unintentionally create an incorrect policy, employing tie strength can limit the effects of the privacy breach by blocking some unwanted audiences.

**Table 3:** Coefficients for the mutual information regression model.

Predictor	*b*
**Tag Used = True**	0.059
**Group Used = True**	0.011
**Ind Used = True**	0.142**
**Tie Used = True**	0.058*

We now build a linear regression model using the type of access control used to define the policy as the predictor and the mutual information as the response variable. Table 4 shows the coefficients yielded by the model. In this case, no coefficient is statistically significant. Thus, there is no evident correlation between the access control employed and the mutual information between an incorrect policy and its corresponding correct one. In other words, the effects of a privacy breach of an incorrect policy are about the same for all access controls.

**Table 4:** Coefficients for the mutual information regression model employing access control type as predictor.

Predictor	*b*
**Access Control = TagGroupIndTie**	−0.027
**Access Control = TagGroupInd**	−0.008

The last analysis to test H-Performance-* hypotheses is to measure the influence of attributes and their combinations on policy redundancies. Sharing policies can include the same contact several times in the allow or block predicates. For example, a user can block a contact from seeing a photo by blocking the group that the contact belongs to and by setting a tie strength threshold that is higher than the contact’s. Redundancies measure the number of times each contact was included several times in the block or allow predicate. Values are relative to the number of contacts that a given participant has. Redundancies can be positive or negative depending on the contact being allowed or blocked several times. High numbers of redundancies can lead to verbose policies, which is detrimental to policy definition and maintenance. Since the number of redundancies is a continuous variable, in this analysis, we employ linear regression models. As in the previous models, we employ mixed modeling. Table 5 shows the coefficients that are yielded by the model. The more attributes are used in a sharing policy, the greater the probability of redundancies. Therefore, all coefficients are significant. Furthermore, the attributes that most often cause positive redundancies are tags and tie strength. Consequently, policies defined with tags and/or tie strength tend to have more redundancies (and be more verbose) than those that do not employ these attributes.

**Table 5:** Coefficients for the positive redundancies model.

Predictor	*b*
**Tag Used = True**	0.408**
**Group Used = True**	0.223**
**Ind Used = True**	0.082**
**Tie Used = True**	0.356**

Now we create a linear regression model for negative redundancies. Table 6 shows the output of the model. Again, all coefficients are significant. However, this time, the attributes that most often cause negative redundancies are tie strength and groups.

**Table 6:** Coefficients for the negative redundancies model.

Predictor	*b*
**Tag Used = True**	0.098**
**Group Used = True**	0.1**
**Ind Used = True**	0.057**
**Tie Used = True**	0.16**

Finally, we create two linear regression models employing the access control type as the predictor variable. Tables 7 and 8 show the coefficients for positive and negative redundancies, respectively. According to the coefficients yielded by the model, when employing TagGroupIndTie, users have greater chances of creating policies with redundancies.

**Table 7:** Coefficients for the positive redundancies model employing access control type as predictor.

Predictor	*b*
**Access Control = TagGroupIndTie**	0.469**
**Access Control = TagGroupInd**	0.144**

**Table 8:** Coefficients for the negative redundancies model employing access control type as predictor.

Predictor	*b*
**Access Control = TagGroupIndTie**	0.2**
**Access Control = TagGroupInd**	0.029

### 4.4 *H-Preference*-*

To evaluate H-Preference-* hypotheses, we employ participant’s responses about which access control they preferred. TagGroupIndTie was chosen by 65.63% of those who tried it, the participants who tried GroupIndTie chose it as the best 53.12% of the time. The least preferred was TagGroupInd, which was chosen 31.25% of the time. Since each participant was assigned two access controls in the study, they chose their preferred access control over the other. Table 9 shows the preferred percentage in pairs. Each row in the table shows the percentage of times that the access control was chosen as the best over the other access control in the column.

**Table 9:** Percentage of times each access control was preferred over each other.

	**TagGroupIndTie**	**TagGroupInd**	**GroupIndTie**
**TagGroupIndTie**	×	68.75%	62.5%
**TagGroupInd**	31.25%	×	31.25%
**GroupIndTie**	37.5%	68.75%	×

We employ a multinomial logistic regression to determine what demographic factors influence the preferred access control the most. Table 10 shows the coefficients yielded by the regression employing the preferred access control as the response variable, and using GroupIndTie as the reference category. Most of the variables are not statistically significant or have low coefficients. There are two exceptions: Facebook experience and whether or not the user has changed her default sharing policies. On the one hand, experienced users are inclined to choose GroupIndTie. On the other hand, users that have changed their default sharing policies on their Facebook accounts prefer TagGroupIndTie over GroupIndTie and prefer GroupIndTie over TagGroupInd.

**Table 10:** Regression coefficients for demographic variables.

	TagGroupIndTie	TagGroupInd
**Gender = Woman**	−1.501	−0.467
**Age**	−0.877	−1.242
**Education level**	−2.772	−2.775
**Facebook experience**	−19.94**	−20.675**
**Facebook frequency of use**	−0.309	−0.05
**Number of friends on Facebook**	−0.644	−0.891
**Number of photos on Facebook**	−2.022*	−1.742
**Privacy concerns on social media**	−2.815*	−2.566
**Default sharing policies changed = Yes**	−41.512**	−50.729**
**Friend lists created = Yes**	−0.134	−0.465
**Knowledge of Facebook data policy**	−1.602	−1.478
**Frequency of sharing policy setting**	−1.104	−1.391

To further explore the preferences of the participants, we classify the given explanations based on the explicit mention of tie strength and tag attributes. Tie strength was the explicit reason why 31.25% of the participants chose an access control over the other. The majority of these participants highlighted the granularity that tie strength provides when classifying contacts. For example, participant P12 said *“It is easier to use. In a given group you may have contacts with high and low proximity, which would require splitting that group into smaller subgroups to create appropriate sharing policies”*. Some participants also indicate that tie strength makes it easier to manage new contacts that have not been assigned to a group yet (P28 *“I think that tie strength is important if you do not have a good set of groups or if you just added somebody and you have not assigned a group to them yet”*).

The participants mentioned explicitly that tags were the reason for choosing one access control over the other 18.75% of the time. The majority of these selections were justified by the simplification that tags provide when defining sharing policies. For example, P1 explained: “*In the first access control, photo categories speed up the whole process. It requires careful organization, though.*”. Some participants also valued the tag view and its organizative functionality (P21 “*I prefer this access control because it offers two perspectives. Of these two perspectives, I like tag the most because it enables me to: 1) organize my photos by category, and 2) since I usually assign the same [privacy] preferences to photos in the same category, it is faster.*)”

We find that 9.25% of the participants disliked tags. These participants considered tags only as a mechanism to organize pictures and failed to see how this attribute could help them to manage their privacy. Finally, we find only one participant that disliked tie strength. This participant pointed out that assigning tie strength values to every contact was a time-consuming task: “*In practice, we do not have that much time to consider who can and who cannot see your photos; it is too time consuming. I think grouping contacts is enough; there is no need for tie strength.*”

## 5 Discussion

The results show that tags and tie strength are extensively employed by users. Therefore, users seem to find that these two attributes are useful in defining their sharing policies. Furthermore, analyzing the self reported preferences of the participants, we find that access controls that consider the attribute tie strength are preferred by users. It is worth noting that 30% of the participants mentioned tie strength as the reason why they chose one access control over the other. Finally, we find that users who are experienced on Facebook and have at least modified the default privacy settings of Facebook prefer TagGroupIndTie and GroupIndTie over TagGroupInd.

When individually analyzing the effects of tie strength and tags on the correctness of sharing policies, we find that tag is the attribute that increases the most the risk of defining an incorrect policy. A study on how combinations of attributes impact policy correctness shows that users create the majority of incorrect policies when combining tie strength and tags. This highlights the fact that when users create complex policies, they find it difficult to foresee the implications of the combinations of these two attributes on the potential viewers. However, we also find that when an incorrect policy is created, tie strength can help to increase the similarity between an incorrect policy and its correct counter part, thus, reducing reduce the seriousness of the privacy breach. We posit that, since every contact has an assigned tie-strength value, this attribute functions as a safe default that prevents acquaintances from accessing sensitive photos.

Finally, we find that tie strength and tags do not increase the ability of users to define succinct policies with few redundancies. However, group attribute has a similar effect on the number of redundancies of sharing policies.

In general, the results show a disconnection between the performance of the attributes and the usage and preferences of the users. On the one hand, the metrics employed to evaluate attributes and access controls indicate that tie strength and tags do not perform as well as the previous literature had anticipated. On the other hand, users intensively employ these two attributes when defining sharing policies and they prefer an access control that offers them, specifically, users that are experienced on Facebook and its privacy controls.

### 5.1 Thematic analysis

To discover the reasons why users prefer access controls with tags and tie strength together, we perform a thematic analysis [32] on the explanations that the participants gave when choosing their preferred access control. This form of analysis goes beyond simply counting phrases or words in a text and moves on to identifying implicit and explicit ideas within the data. To that aim, we define three categories that classify the impressions of the participants:

- **Understandability**: Participants valued how easy it was to comprehend who will be allowed to see each photo and who will not. An example of this class is: “*This access control is easier to manage. There may be people in the same group that have different degrees of closeness, so you need to be careful and pick them individually. However, by employing tie strength, you know, for sure, that only very close people can see the photo.*”.
- **Granularity**: A number of participants perceived that some access controls offered finer levels of granularity that enabled them to carefully tailor who could view their personal information. An example of this class is: “*I prefer the second access control because I can make a better selection of who can and who cannot see my photos. It not only allows me to choose a group, but also to include some people from another group or remove some from the selected group so they cannot see the photo.*”
- **Speed of configuration**: While some participants preferred to have fine-grained access controls, other were more satisfied with access controls that can be set quickly. An example is: “*Access control two requires too much time. It is faster to create small groups and categorize the photos.*”

Overall, the classification of explanations was as follows: understandability (26.31%), granularity (28.95%), speed of configuration (44.74%). With these categories, 75% of the explanations were classified. Those that could not be classified were either too vague or the participant failed to clearly express their perception. Categories were not exclusive, thus, an explanation could be classified into two or more classes.

Table 11 shows the explanation class spread for all of the access controls. An interpretation of the results indicates that the participants considered that it is easier to understand the consequences of a sharing policy defined with an access control that combines tie strength and tags. They also considered that tie strength offers high levels of granularity when defining sharing policies. Furthermore, the participants valued the speed that tags offer; enabling them to set sharing policies faster.

**Table 11:** Spread of explanation classes across access controls.

	TagGroupIndTie	TagGroupInd	GroupIndTie
**Understandability**	70%	20%	10%
**Granularity**	45.46%	18.18%	36.36%
**Speed**	23.53%	47.06%	29.41%

The results obtained in this study generalize to other Facebook-like SNSs, such as Google+ or LinkedIn. However, other SNSs (e.g.,Twitter or Instagram) that are focused on other social aspects will need specific studies to evaluate what new attributes can improve their access controls.

### 5.2 Practical implications

According to the results, users value the addition of tie strength and tags positively, however, the results also indicate that users will require assistance when using these attributes. Furthermore, adding new attributes to an access control can increase the effort that users have to dedicate to set their privacy preferences. However, there are a number of automated approaches that can alleviate these problems. We envision an access control that offers attributes that users can employ to effectively define their privacy preferences while still being easy and quick to set up. To illustrate how a user could deal with these new attributes, imagine an SNS that offers the following functionalities:

- **Automatic contact grouping and tie strength computation**: Considering that the average number of contacts Facebook users have is around 160 [33], grouping contacts and specifying a tie strength value for each one can be a daunting task. Therefore, SNSs should offer tools that alleviate this task. A number of research works propose tools that can help users group their contacts [21, 23]. Similarly, tools such as BFF [27] can predict tie strength with acceptable accuracy.
- **Automatic photo tag inference**: It can be expected that each user habitually shares the same types of photos. A tool such as the one proposed by Kucuktunc [34] can learn tags from previous photos shared by a user and tag new photos accordingly. Therefore, users would only need to specify tags for a few initial photos. New photos would automatically be tagged, therefore speeding up the process of defining sharing policies employing tags.
- **Personalization**: Current access controls for SNS are static; they do not adapt to users. However, as the qualitative evaluation shows, experience with social media influences the choice of access control. We posit that users should be able to configure the attributes employed by the access control. In this way, as users get accustomed to how photo sharing works on SNS and its implications, they could adapt the access control to meet their specific needs.
- **Sharing policy assistant**: The results obtained by tie strength and tags on the correctness test and by all attributes on the redundancy test highlight that users require assistance when defining sharing policies. A visualization tool similar to the one proposed by Lipford et al. [35] can help users understand the implications of a sharing policy. Similarly, a misconfiguration detection utility, such as [36], could indicate to users which sharing policies have more chances of being incorrect. Finally, to reduce the redundancy of policies, the SNS could offer a policy simplification tool such as the one presented in [37].

Building users’ trust in automatic photo tagging, contact grouping, and tie-strength computation is key for the success of new access controls with new attributes. To this aim, SNSs should introduce these elements as recommendations that require the user’s supervision and, as the user becomes more accustomed with automation, the user’s supervision would only be required when the automatic inference is unsure about the result.

### 5.3 Limitations

There are a number of limitations to our study design. First, our results are limited by the participants that we recruited and the photos that they provided. As shown in Demographics, the sample of participants was skewed towards young people with a high level of education. Additionally, the population was homogeneous in terms of location. The majority of participants were recruited from the same area in Spain.

We asked the participants to provide photos that fulfilled a list of 17 requirements. However, the list may not be comprehensive enough and not cover every type of photo that users normally upload to an SNS. Therefore, the generalizability of the photos provided, which may affect the results obtained by the attributes and access controls, was also limited to what the participants considered appropriate.

Other limitations concern scale. We cannot comment on whether all of access controls and the two new attributes would remain viable when dealing with thousands of photos and hundreds of contacts of varying types (friends, family members, and acquaintances). Further work should be done in this regard.

Finally, our study is cross-sectional (one time). Our study does not provide data to understand how users deal with access controls and their attributes over a period of time. This might be the reason why, even though users like tie strength and tags, these two attributes did not perform as well as the more familiar group and individual attributes. It may be that for users to obtain the full benefit from these two new attributes, they need to iteratively refine tie strength values and tags as they learn the effects of these attributes on their sharing policies.

## 6 Conclusions

Social media has become one of the most common and useful ways of sharing photos. Nonetheless, unsatisfactory privacy management in SNS leads to privacy leaks that may drive users away from employing all the functionalities offered by SNSs. In order to improve privacy management in SNSs, developers have to offer access controls that are easy to understand and that make users feel that they are in control of how their information is disseminated. Related literature had identified tie strength and tags as attributes with potential to improve access controls. In this paper, we evaluate their viability.

An analysis of the preferences and use of each attribute indicates that users value positively tie strength and tags and find them useful in defining sharing policies. Moreover, a qualitative analysis informs about what features users find important in an access control. Based on this, users value the granularity and understandability that tie strength offers and how tags can speed up the privacy configuration process. Nonetheless, when users employ these two new attributes, they tend to make more mistakes in terms of policy correctness. Nonetheless, tie strength can help reduce the damage that a privacy breach may cause. We hypothesize that the results obtained by tie strength and tags in terms of policy correctness and redundancy can point to a lack of experience dealing with these attributes in an access control. Therefore, SNS developers who are willing to include these attributes should consider first the addition of tools and mechanisms that help users understand the implications of tie strength and tags on their privacy, at least, until users become accustomed to their use. Future work should test whether users that are educated about how to use tie strength and tags make fewer mistakes than those who use them for the first time.

## Supporting information

S1 Appendix Post-Survey questionnaire in Spanish. (PDF)

S2 Appendix Post-Survey questionnaire in English. (PDF)
