# Supplementary material for: Tie and tag: A study of tie strength and tags for photo sharing
Source: PLoS One. 2018 Aug 29;13(8):e0202540. doi: 10.1371/journal.pone.0202540 (PMC6115014; doi:10.1371/journal.pone.0202540)
Supplement: S1 Appendix — (PDF) [file pone.0202540.s001.pdf]

Inscripción experimento GTI IA sobre privacidad en Facebook

*Required

1. **Sexo** *

Mark only one oval.

- Mujer
- Hombre

2. **Edad** *

Mark only one oval.

- 18 - 24
- 25 - 29
- 30 - 39
- 40 - 49
- 50 - 59
- 60+

3. **Estudios** *

Mark only one oval.

- ESO / Graduado escolar
- Bachiller
- Universitario
- Doctorado

4. **¿Cuál es su ocupación actual?** *

Mark only one oval.

- Estudiante
- Trabajador por cuenta ajena
- Trabajador por cuenta propia
- Desempleado

5. **¿Qué antigüedad tiene su perfil en Facebook?** *

Mark only one oval.

- Menos de un año
- Entre uno y dos años
- Más de dos años

6. **¿Con que frecuenca usa Facebook?** *
Mark only one oval.

- ( ) Varias veces al día
- ( ) Una vez al día
- ( ) Una vez cada pocos días
- ( ) Una vez a la semana
- ( ) Una vez al mes
- ( ) Menos de una vez al mes

7. **Indique cuales son las acciones habituales que realiza en Facebook** *
Tick all that apply.

- [ ] Mirar información sobre un amigo
- [ ] Hablar con amigos
- [ ] Subir fotos
- [ ] Mirar fotos subidas por otros
- [ ] Publicar historias o links
- [ ] Encontrar amigos

8. **¿Cuántos amigos tiene en su cuenta de Facebook?** *
Mark only one oval.

- ( ) 0 - 49
- ( ) 50 - 100
- ( ) 100 - 199
- ( ) 200 - 399
- ( ) 400 - 599
- ( ) 600+

9. **¿Cuántas fotos tiene en su perfil de Facebook?** *
Mark only one oval.

- ( ) Menos de 10
- ( ) Entre 10 y 50
- ( ) Ente 50 y 100
- ( ) Más de 100

10. **En general ¿se considera una persona preocupada por su privacidad en Internet?** *
Mark only one oval.

- ( ) Extremadamente
- ( ) Mucho
- ( ) Moderadamente
- ( ) Ligeramente
- ( ) Nada

11. **¿Ha modificado la configuración por defecto de privacidad de Facebook?** *

Mark only one oval.

- Sí
- No

12. **¿Ha usado la utilidad para crear grupos de contactos en Facebook?** *

Mark only one oval.

- Sí
- No

13. **¿Como calificaría su conocimiento sobre la cantidad y la forma en que su información se comparte en Facebook?** *

Mark only one oval.

- Extremadamente bueno
- Muy bueno
- Moderadamente bueno
- Ligeramente bueno
- Malo

14. **¿Con que frecuencia suele asignar una configuración de privacidad a sus publicaciones o fotos?** *

Si siempre asigna una configuración de privacidad quiere decir que siempre escoge grupos o individuos los cuales podrán acceder a la publicación. Si no usa nunca las configuraciones de privacidad es porque siempre escoge la opción por defecto que le ofrece Facebook.

Mark only one oval.

- Siempre
- Casi siempre
- Esporádicamente
- Nunca

15. **¿Alguna vez se ha desetiquetado de una foto subida por un amigo suyo?** *

Mark only one oval.

- Sí
- No

Datos personales

16. **Nombre** *

17. **Apellidos** *

18. **Nombre perfil Facebook** *

19. **Dirección de e-mail** *

Powered by
Google Forms
