# Supplementary material for: Tie and tag: A study of tie strength and tags for photo sharing
Source: PLoS One. 2018 Aug 29;13(8):e0202540. doi: 10.1371/journal.pone.0202540 (PMC6115014; doi:10.1371/journal.pone.0202540)
Supplement: S2 Appendix — (PDF) [file pone.0202540.s002.pdf]

Registration for GTI IA Facebook privacy study

*Required

1. **Gender** *

   Mark only one oval.

   - Female
   - Male

2. **Age** *

   Mark only one oval.

   - 18 - 24
   - 25 - 29
   - 30 - 39
   - 40 - 49
   - 50 - 59
   - 60+

3. **Study level** *

   Mark only one oval.

   - School
   - High School
   - Undergrad
   - Masters or PhD

4. **Occupation** *

   Mark only one oval.

   - Student
   - Employed
   - Self-employed
   - Unemployed

5. **How old is your Facebook account?** *

   Mark only one oval.

   - Less than a year
   - Between one and two years
   - More than two years

6. **How often do you use Facebook?** *
Mark only one oval.

- Several times a day
- Once a day
- Once every few days
- Once a week
- Once a month
- Less than once a month

7. **Mark the actions that you usually do on Facebook** *
Tick all that apply.

- Look for information about a friend
- Talk with friends
- Upload photos
- Look at photos uploaded by others
- Post stories and links
- Look for friends

8. **How many friends do you have on Facebook?** *
Mark only one oval.

- 0 - 49
- 50 - 100
- 100 - 199
- 200 - 399
- 400 - 599
- 600+

9. **How many photos do you have on Facebook?** *
Mark only one oval.

- Fewer than 10
- Between 10 and 50
- Between 50 and 100
- More than 100

10. **In general, do you consider yourself as a person worried about your privacy on the Internet?** *
Mark only one oval.

- Extremely
- Verymuch
- Moderately
- Lightly
- Not at all

11. **Have you ever modified the default privacy configuration of Facebook?** *
Mark only one oval.

- Yes
- No

12. **Have you ever created groups on Facebook?** *
Mark only one oval.

- Yes
- No

13. **How would you evaluate your knowledge about the information Facebook collects from you and and how it is managed?** *
Mark only one oval.

- Extremely good
- Very good
- Moderately good
- Not so good
- Bad

14. **How often do you assign a sharing policy to your publications on Facebook?** *
If you always assign a sharing policy it means that you always choose the individuals and groups that can access the publication. If you never assign a sharing policy, it means that you always use the default privacy configuration offered by Facebook.
Mark only one oval.

- Always
- Almost always
- Sometimes
- Never

15. **Have you ever untagged yourself from a photo uploaded by a friend of yours?** *
Mark only one oval.

- Yes
- No

Personal information

16. **Name** *

17. **Surnames** *

18. **Facebook profile name** *

19. **E-mail address** *

Powered by
Google Forms
